# Enhanced sprint performance analysis in soccer: New insights from a GPS-based tracking system

**DOI:** 10.1371/journal.pone.0217782

**Published:** 2019-05-31

**Authors:** Lars Reinhardt, René Schwesig, Andreas Lauenroth, Stephan Schulze, Eduard Kurz

**Affiliations:** Department of Orthopedic and Trauma Surgery, Martin-Luther-University Halle-Wittenberg, Halle (Saale), Germany; Universidade Federal de Juiz de Fora, BRAZIL

## Abstract

The aim of this investigation was to establish the validity of a GPS-based tracking system (Polar Team Pro System, PTPS) for estimating sprint performance and to evaluate additional diagnostic indices derived from the temporal course of the movement velocity. Thirty-four male soccer players (20 ± 4 years) performed a 20 m sprint test measured by timing gates (TG), and while wearing the PTPS. To evaluate the relevance of additional velocity-based parameters to discriminate between faster and slower athletes, the median-split method was applied to the 20-m times. Practical relevance was estimated using standardized mean differences (d) between the subgroups. Differences between the criterion reference (TG) and PTPS for the 10 and 20 m splits did not vary from zero (dt10: -0.01 ± 0.07 s, P = 0.7, d < -0.1; dt20: -0.01 ± 0.08 s, P = 0.4, d < -0.2). Although subgroups revealed large differences in their sprint times (d = -2.5), the average accelerations between 5 and 20 km/h as well as 20 and 25 km/h showed merely small effects (d < 0.5). Consequently, analyses of velocity curves derived from PTPS may help to clarify the occurrence of performance in outdoor sports. Thus, training consequences can be drawn which contribute to the differentiation and individualization of sprint training.

## Introduction

As in most team sports, soccer is about scoring and preventing goals, whereas straight sprinting with and without the ball was found to occur in approximately every second goal situation in the first German national league [1]. Since all players on the pitch are involved in these situations, sprinting is of outstanding importance and thus a crucial element of the requirement profile. This estimation was also shared by many authors [2–5], although the cumulative sprint distance is below 5% relative to the total distance covered during a match. Furthermore, the vast majority of sprint displacements are below 20 m [6–9]. Moreover, investigations in the English Premiere League across seven seasons from 2006/07 to 2012/13 showed a massive increase in the distance covered in the high-intensity (24–36%) and sprinting (36–63%) zone in all playing positions although the total distance only changed marginally [2, 3]. Following common conventions, high-speed running and sprinting are achieved when at least 20 or 25 km/h are reached, respectively [3, 7, 10–12]. As a matter of course, every sprint consists of an initial acceleration phase in order to gain running velocity. Nevertheless, accelerating is more energetically demanding than moving at constant velocity [13]. In spite of an 8-fold higher number of maximal accelerations than sprints per game, this phase is frequently excluded from analysis, since the high-intensity running threshold is not crossed [12].

To manage training intensities appropriately, performance diagnostics are required. In this context, special technologies are commonly utilized. The most widely applied and proven technology involves photo-electric timing gates (TG) [14]. However, TG solely measure the time required to cover a given distance and therefore can only be used to calculate average velocities within sections [15]. Since velocity profiles are leading to more meaningful information (i.e. peak velocity, initial acceleration, etc.), different technologies need to be involved. At this point, GPS (global positioning system)-based tracking systems are frequently considered in team sports [16, 17]. In recent years, this technology has become a standard tool for routine load monitoring in training and competition [18]. The validity and reliability of GPS devices has already been proven extensively (e.g. [19, 20, 21]). While radar guns served as the criterion measure, the velocity traces were found to be accurate during acceleration, deceleration as well as constant movements [21]. As mentioned earlier, the main advantage of these devices in the context of sprint performance diagnostics is that they are able to measure the temporal course of the velocity. Additionally, the possibility of measuring multiple players synchronously is opened up and there are no local restrictions due to a fixed pathway (as in the case of TG), so that multidirectional movements or curved runs could also be investigated.

Against this background, we assume that parameters derived from the time course of the running velocity determined by a GPS-based tracking system represent an added value for sprint diagnostics compared to split times measured by TG. Thus, this investigation aimed at analysing velocity profiles during linear short-distance sprinting while TG served as the criterion reference to validate times and distances determined via GPS. In addition, velocity profiles were used to parametrize sprint performance section-wise.

## Methods

### Participants and tasks

One linear sprint per athlete over 20 m distance was concurrently examined via TG and a wearable motion sensor in a population of 34 male outfield soccer players (age: 20 ± 4 years; mean ± SD; height: 180 ± 6 cm; mass: 74 ± 7 kg; BMI: 23.0 ± 1.4 kg/m^2^) on artificial turf. The sample consisted of two separate teams of a German soccer club: 13 members of the professional (PRO) and 21 players of the under-19 team (U19). The study protocol used was approved by the Martin-Luther-University Halle-Wittenberg Institutional Review Board (IRB # 2013–13), and all participants agreed to enter the study after having been informed orally on study details.

All athletes performed the FIFA 11+ routine as a standardized warm-up complemented with specific speed drills prior to testing. A rest of approximately three minutes between warm-up and test was administered to avoid fatigue effects. Further, participants were tested at least 48 h after the last intense training to minimize fatiguing effects [15]. In order to record 10 m and 20 m split times (t10, t20) to the nearest 0.01 s, single-beam TG (DLS-LA Timing System, AF Sport, Wesel, Germany) were placed at 0, 10 and 20 m. The sprinting course was sized using a tape measure, and the TG were mounted at a height of one meter (which corresponds to the average hip height of adult males) to prevent them from being triggered too early by raised knees or swinging arms [22]. Following a recent review by Haugen and Buchheit, in a single-beam setup the standard error of measurement is about 0.03 s [14]. Participants started 0.5 m behind the first TG in a forward leaning split-stance position with slightly bended knees and their preferred foot in front. Each participant wore a Polar Team Pro sensor (PTPS, Polar Electro, Kempele, Finland) which recorded spatial position (GPS) and heart rate simultaneously. Thus, the PTPS was attached to the skin (with a commercially available strap) approximately above the xiphoid process. The PTPS starts recording as soon as a heart rate signal is detected and saves data to an internal storage. As recommended by Polar, the electrode area of the straps was moistened with water to achieve an optimal connection. Outdoors, the GPS signal was typically received within a few seconds. The measurements were carried out on a cloudless, precipitation-free late summer day (temperature: ~20° C; relative humidity: 48%; atmospheric pressure: 1002.5 hPa). There were no larger buildings in the immediate vicinity of the test field.

### Data acquisition and processing

**Fig 1:**
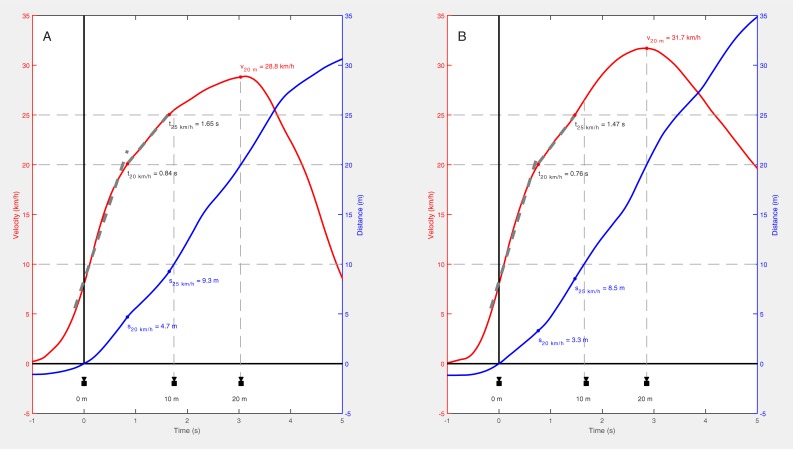
Sample results of a slower (A) and a faster (B) soccer player. Grey dashed lines are linear regressions with slope representing the average acceleration within the two different phases. Camera symbols represent the associated split times measured by the timing gates. See text for details.

As specified by the manufacturer, the PTPS combines signals from a 10 Hz GPS with a 200 Hz microelectromechanical inertial measurement unit (IMU) consisting of a tri-axial accelerometer, gyroscope and magnetometer. Based on these signals, data fusion algorithms are applied within the device to improve spatial accuracy [23]. In principle, the algorithms mentioned are based on sensor fusion of GPS and IMU data using a Kalman filter [24]. Raw data (velocity, distance time series) were exported as CSV files and further processed in MATLAB R2016a (The MathWorks, Natick, MA, USA). No filter was applied to the time series. In order to relate the split times of the TG to the raw signals of the PTPS, a fixed threshold of 8 km/h was set for the moment when passing the first TG (see Fig 1). This specific threshold was chosen due to the known gait transition (walking to running) velocity in human locomotion of between 7 and 8 km/h [25].

### Outcomes

To characterize sprinting performance, several parameters were derived from the velocity and distance trajectories (PTPS) as outlined exemplarily in Fig 1. For PTPS validation purposes, TG results served as the criterion reference [20, 26]. In order to determine *t*_*10*_ and *t*_*20*_ based on PTPS data, the times taken for covering the corresponding distances were calculated. Otherwise, the accuracy of the PTPS distance estimation was determined by relating the distances (*s*_*10*_, *s*_*20*_) covered within the split times of the TG system.

Through the availability of synchronized time series, a velocity-based analysis was performed here. Accordingly, the times (*t*_*20 km/h*_, *t*_*25 km/h*_) and distances (*s*_*20 km/h*_, *s*_*25 km/h*_) for reaching 20 as well as 25 km/h (*v*_*20 km/h*_, *v*_*25 km/h*_) were analyzed separately for each athlete. Furthermore, the velocities when passing the second (*v*_*10 m*_) and third (*v*_*20 m*_) TG were determined.

In addition, the velocity curve between 5 and 20 km/h as well as 20 and 25 km/h, respectively, was fitted by a linear regression. The slopes of the two regression lines (*acc*_*1*_, *acc*_*2*_) represented average accelerations within the respective phases.

### Statistical analyses

Statistical analyses were carried out with MATLAB R2016a (Mathworks Inc., Natick, MA) software. Data distribution was visually inspected and evaluated by skewness, kurtosis and the Shapiro-Wilk’s test. Validity was established by comparisons of the 10 m and 20 m sprint times of the PTPS with those measured by TG using Pearson’s correlation coefficients (*r*) with 95% confidence intervals (CI), whereas the agreement of both measurement systems was inspected with difference plots and limits of agreement. To indicate accuracy, root-mean-square error (RMSE) was calculated [22]. The between-subject variability was assessed using coefficient of variation (CV). Possible effects between teams as well as performance subgroups (median-split, [27]) were verified utilizing Student’s t test for independent samples separately on each parameter. Practical relevance was estimated calculating standardized mean differences (d) with values ≥ 0.2, ≥ 0.5, ≥ 0.8 indicating small, moderate, or large effects, respectively [28].

## Results

### Sprint performance (TG)

The split times measured by TG did not differ between PRO (*t*_*10*_: 1.75 ± 0.08 s, *t*_*20*_: 3.03 ± 0.10 s) and U19 (*t*_*10*_: 1.72 ± 0.07 s, *P* = 0.16, d = 0.51; *t*_*20*_: 2.99 ± 0.10 s, *P* = 0.33, d = 0.35). The variability within each team was low and of comparable magnitude (*t*_*10*_: CV < 4.5%; *t*_*20*_: CV < 3.4%).

### Validity

**Fig 2:**
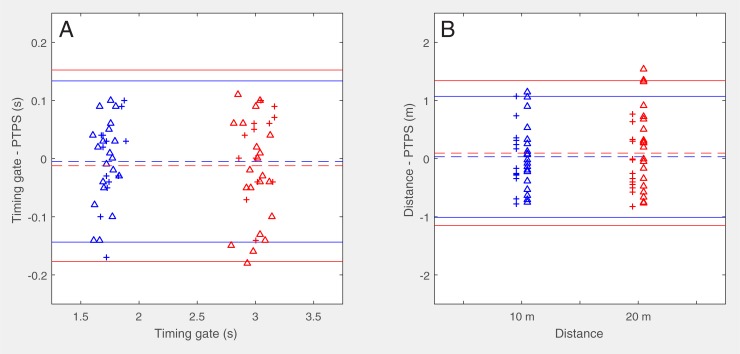
Difference plots with limits of agreement of times (A) and distances (B) of 10 m (blue) and 20 m (red) sprints depicted separately for athletes from the professional team (*n* = 13, crosses) and the under-19 players (*n* = 21, triangles). Dashed lines are means and solid lines represent 95% limits of agreement.

Low (r = 0.57, CI: 0.40–0.86, *P* < 0.001) to moderate (r = 0.74, CI: 0.57–0.91, *P* < 0.001) relationships were found between the results of TG (*t*_*10*_ and *t*_*20*_) and times estimated by PTPS, respectively. Differences between the criterion reference (TG) and PTPS for *t*_*10*_ and *t*_*20*_ did not vary from zero (*dt*_*10*_: -0.01 ± 0.07 s, *P* = 0.7, d = -0.07; *dt*_*20*_: -0.01 ± 0.08 s, *P* = 0.4, d = -0.15). Neither did the differences vary between teams (PRO: *dt*_*10*_: -0.01 ± 0.08 s, *dt*_*20*_: 0.01 ± 0.07 s, *P* > 0.9, -0.08 < d < 0.20; U19: *dt*_*10*_: 0.00 ± 0.07 s, *dt*_*20*_: -0.03 ± 0.09 s; *P* = 0.16, -0.33 < d < -0.07). Consequently, the comparisons of the results between measurement systems with difference plots (Fig 2) were performed with all participants tested. RMSE was 0.07 s for *t*_*10*_ and 0.08 s for *t*_*20*_, respectively.

### Sprint performance categorization (median-split of 20 m times)

**Fig 3:**
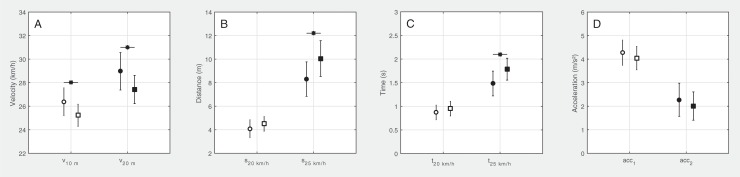
Characteristic values of GPS-based time series between faster (FA, *n* = 17, circles) and slower (SL, *n* = 17, squares) soccer players with significant differences at the 0.01 level marked with an asterisk. Values are means with standard deviations. Please refer to the text for a detailed description of the parameters used.

The *t*_*20*_ measured by TG revealed a normal distribution (Shapiro-Wilk’s test, *P* > 0.4, skewness: -0.28, kurtosis: -0.44). Thus, the median-split method was applied to categorize the short-distance sprint performance into a faster (FA, *n* = 17, PRO: 7, U19: 10) and a slower (SL, *n* = 17, PRO: 6, U19: 11) subgroup based on 20 m sprint times derived from timing gates (see Fig 3).

### Subgroup comparisons of PTPS parameters

As expected, characteristic velocities showed strong differences between FA and SL (d > 1.0, Fig 3A). The distances after which the subgroups reached 20 or 25 km/h revealed moderate (d = -0.6) to large (d = -1.2) differences (Fig 3B). The PTPS-based times revealed large differences for the instant where subgroups reached 25 km/h (d = -1.2, Fig 3C). Although the instants where 20 km/h was achieved showed moderate differences between subgroups (d = -0.5), this result did not reach statistical significance (*P* > 0.1). The average accelerations determined by linear regressions showed only small differences between categorized subgroups (*P* > 0.2, d < 0.5, Fig 3D).

## Discussion

This study investigated the validity of the PTPS to estimate short-distance linear sprint performance using velocity profiles. The analyses of velocity curve progressions enabled a section-wise evaluation. Within the distance of eight to ten meters, two characteristic slopes of the velocity curve were identified. These phases represented different average accelerations which could not predict the sprint time over 20 m distance.

The sprint times measured in our samples were comparable to those of previous studies on professional soccer players [29–31] or U19 athletes [29, 32, 33]. Consequently, the analyses presented here can be regarded as generally applicable in elite soccer. The longer the sprint distance measured, the lower the between-subject variability, which most probably results from different photocell triggers within slower locomotion velocities [14]. This fact is frequently discussed as a limitation in applying timing gates to monitor performance in field sport athletes.

As pointed out by Akenhead and colleagues [34], the accuracy of GPS measures is compromised at higher accelerations (> 4 m/s^2^). This is in line with our results (see Fig 3D) and explains, in part, the lower relationships with the criterion reference within the early acceleration phase which is defined by the 10-m time (*t*_*10*_) in short-distance sprints [15]. However, the results of the correlation analysis were most likely much more affected by the restricted range of values due to the homogenous sample. The range of the 20-meter times was 0.37 s and thus about 30% higher than that of the 10-meter times (0.29 s). Correspondingly, the correlation was higher in *t*_*20*_ than in *t*_*10*_. Based on our results, the measurement errors of the tracking system used for the sprint times and distances were below 0.18 s or 1.35 m, respectively (Fig 2). As reported by different previously conducted studies on 10 Hz GPS devices, validity increased with increasing sprint distances [20, 35]. Numerous studies already examined the validity of kinematic data derived from GPS devices against timing gates [35–37]. For an extensively performed review on validity and reliability of GPS-based devices, please refer to Scott and colleagues [38].

**Fig 4:**
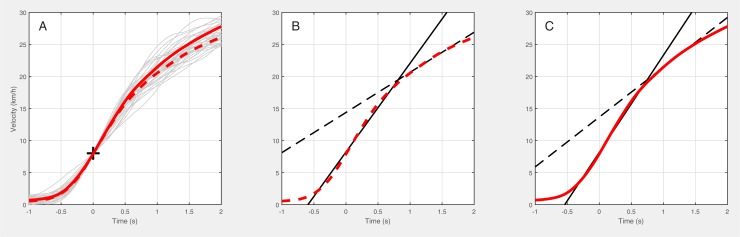
Mean velocity profiles of slower (SL, *n* = 17, solid red line) and faster (FA, *n* = 17, dashed red line) soccer players, all with individual profiles (A). The cross indicates the predefined threshold at 8 km/h. Mean velocity profiles of SL (B) and FA (C) are illustrated with the corresponding average accelerations (acc, black line) within the two distinguished phases (*acc1*, solid line; *acc2*, dashed line).

Due to the measuring principle which is leading to a limited number of sampling points, timing gates provide only average velocities. Analyses of velocity profiles measured by wearables in turn overcome this limitation. In the present study, each athlete investigated reached the velocity of 20 km/h (high-speed running) before a travelled distance of 6 m, whereas the sprinting threshold (25 km/h) was achieved between 6 and 13 m. In this way, average accelerations of up to 5 m/s^2^ were achieved within the first half of the sprint (Fig 4D). The ability to accelerate and decelerate their body mass from various velocities (rarely standing still) is of fundamental importance for soccer players [15]. For example, a player has to produce high accelerations to quickly enlarge the distance to an opponent in offensive actions or to reduce the distance in the defense, respectively [39]. Since the sprint performance categorization (median-split, [27] was applied via the 20 m splits, which corresponds to more than 80% of athletes’ maximum velocity [40], parameters related to performance characteristics (*v*_*10 m*_, *v*_*20 m*_, *s*_*25 km/h*_, *t*_*25 km/h*_) above 6 m differed at least moderately between subgroups. Murphy and colleagues [27] found that the early acceleration phase lies within the distance between 5 and 10 m. In short-distance linear sprints, it is widely accepted that *t*_*10*_ represents the athletes’ ability to accelerate [15, 41–43]. However, although there is no consensus on determination of an acceleration threshold [44] within the early acceleration phase, two independent phases were found in this study. The results of these two acceleration phases failed to distinguish between the faster and slower athletes. While *acc*_*1*_ includes the first steps up to 6 m, *acc*_*2*_ comprises later acceleration abilities (see Fig 4).

The mechanical differences between distinct sprint phases within a 20 m straight-line sprint were impressively highlighted after specific resistance training interventions [45, 46]. These studies emphasized the presence of early (initial) and later acceleration subphases in short-distance sprint efforts. To reduce training errors and optimize training adaptations, Brown and colleagues [47] differentiated between three consecutive short-distance sprint phases in team sport athletes.

Interestingly, only small differences in *acc*_*1*_ as well as *acc*_*2*_ were found between categorized subgroups. This is most probably due to the higher within-subject variability (CV), which indicates athletes’ different performance predispositions within both acceleration phases. Thus, the parameters mentioned can help to better distinguish between adequate exercises to improve different acceleration attributes (e.g. start, first-step quickness, initial acceleration, pick-up acceleration) and thus systematically improve the global sprint performance. Moreover, the different characteristics of acceleration suggest a probable association of *acc*_*1*_ with attributes of agility, conversely to the three distinct physical attributes of speed of movements (agility, acceleration, top speed), as pointed out by Little and Williams [15].

The methods applied in this study also have some limitations that need to be addressed. First, the fact that the split times did not differ between PTPS and TG is a strong indication, but not proof, of validity of the velocity curves. Undoubtedly, radar guns would have been better suited for this purpose [48]. Furthermore, the performed synchronization of the two measuring systems at a fixed velocity of 8 km/h can be criticized, but was at least in our opinion the best solution, since no interface existed between the two measurement systems. The fact that this approach was successful in the present study does not necessarily mean that it can also be applied to differing test situations (especially with higher starting velocities). Finally, given the fact that the two acceleration phases discovered took place in the first half of the sprint distance, the concurrent use of motion capture systems (e.g. Vicon, Qualisys) or high-speed video cameras could have been considered. This approach could have provided additional information about differences in running technique.

## Conclusion

In conclusion, the results of this study indicate that velocity curves derived from a GPS-based tracking system are a valid and useful database to analyze sprint performance. Especially the section-wise calculation of kinematic parameters in defined velocity ranges leads to additional perspectives on the occurrence of sprint performance in outdoor sports. Thus, specific training consequences can be drawn which contribute to the differentiation and individualization of short-distance sprint training. Due to the fact that these technologies are used by the majority of the elite soccer teams on a daily basis, it is reasonable to use the data captured as a source of additional information concerning the sprint performance. This compensates for the disadvantage of existing measurement inaccuracy.

## Supporting information

S1 File Raw dataset. (XLSX)
